# Pancreatic stone protein as a novel marker for neonatal sepsis

**DOI:** 10.1186/cc11765

**Published:** 2012-11-14

**Authors:** L Schlapbach, R Graf, A Woerner, M Nelle, M Stocker, M Fontana, U Zimmermann-Baer, D Glauser, E Giannoni, T Roger, C Mueller

**Affiliations:** 1 Mater Children's Hospital, South Brisbane, Australia; 2 Swiss HPB Center, University Hospital Zurich, Switzerland; 3 University of Berne, Switzerland; 4 Children's Hospital Lucerne, Switzerland; 5 Clinic of Neonatology, Cantonal Hospital Winterthur, Switzerland; 6 Service of Neonatology, Centre Hospitalier Universitaire Vaudois and University of Lausanne, Switzerland; 7 Infectious Diseases Service, Centre Hospitalier Universitaire Vaudois and University of Lausanne, Switzerland; 8 Institute for Pathology, Universiy of Berne, Switzerland

## Background

Early-onset sepsis (EOS) represents one of the main causes for ICU admission in newborns and therefore imposes a considerable burden on the healthcare system. Performance of traditional infection markers to diagnose EOS is poor and therefore insufficient to guide the decision to start or stop antibiotic treatment. While procalcitonin (PCT) has become an established sepsis marker in adults and children, sensitivity and specificity of PCT are only moderate in EOS due to the physiologic PCT increase during the first days of life. Pancreatic stone protein (PSP) is a promising sepsis marker in adults. This study investigated whether determining PSP improves diagnosis of EOS in comparison with other infection markers.

## Methods

A prospective multicentre study including 137 infants >34 weeks gestational age admitted with suspected EOS. PSP, PCT, soluble human triggering receptor expressed on myeloid cells-1 (sTREM-1), macrophage migration inhibitory factor (MIF) and C-reactive protein (CRP) were measured at admission. Receiver-operating characteristics (ROC) curve analysis and multivariate logistic regression were performed. Bioscores were constructed using two, three and four sepsis markers.

## Results

PSP was significantly higher in infected compared with uninfected infants (median 11.3 vs. 7.5 ng/ml, *P *= 0.001). The ROC area under the curve resulted at 0.69 (95% CI = 0.59 to 0.80, *P *< 0.001) for PSP, at 0.77 (95% CI = 0.66 to 0.87, *P *< 0.001) for PCT, at 0.66 (95% CI = 0.55 to 0.77, *P *= 0.006) for CRP, at 0.62 (0.51 to 0.73, *P *= 0.055) for sTREM-1 and at 0.54 (0.41 to 0.67, *P *= 0.54) for MIF. In multivariate models, increased PSP levels showed the strongest association of all markers with EOS and PSP >9 ng/ml independently of PCT predicted EOS (odds ratio = 26.4, 95% CI = 4.0 to 172.5, *P *< 0.001). Combining both markers significantly increased the ability to diagnose EOS (*P *< 0.001). The bioscore based on PSP and PCT performed best with an AUC of 0.83 (95% CI = 0.74 to 0.93, *P *< 0.001) and was superior to PCT or PSP alone. The combined PSP/PCT score had a NPV of 100% if both markers were below cutoff and a PPV of 71% if both were positive.

## Conclusion

In this prospective study, the diagnostic performance of PSP and PCT was far superior compared with traditional markers and a combination bioscore improved diagnosis of sepsis. Our findings suggest that PSP is a valuable biomarker in combination with PCT in EOS. To the best of our knowledge, this is the first study investigating PSP in neonatal sepsis.

